# Structural Pathways between Child Abuse, Poor Mental Health Outcomes and Male-Perpetrated Intimate Partner Violence (IPV)

**DOI:** 10.1371/journal.pone.0150986

**Published:** 2016-03-17

**Authors:** Mercilene T. Machisa, Nicola Christofides, Rachel Jewkes

**Affiliations:** 1 Gender and Health Research Unit, South African Medical Research Council, Pretoria, South Africa; 2 School of Public Health, University of Witwatersrand, Johannesburg, South Africa; University of Illinois-Urbana Champaign, UNITED STATES

## Abstract

**Background:**

Violent trauma exposures, including child abuse, are risk factors for PTSD and comorbid mental health disorders. Child abuse experiences of men exacerbate adult male-perpetrated intimate partner violence (IPV). The relationship between child abuse, poor mental health and IPV perpetration is complex but research among the general population is lacking. This study describes the relationship and pathways between history of child abuse exposure and male-perpetrated IPV while exploring the potentially mediating effect of poor mental health.

**Methods:**

We analysed data from a randomly selected, two-stage clustered, cross-sectional household survey conducted with 416 adult men in Gauteng Province of South Africa. We used multinomial regression modelling to identify associated factors and Structural Equation Modelling (SEM) to test the primary hypothesis that poor mental health (defined as abusing alcohol or having PTSD or depressive symptoms) mediates the relationship between child abuse and IPV perpetration.

**Results:**

Eighty eight percent of men were physically abused, 55% were neglected, 63% were emotionally abused and 20% were sexually abused at least once in their childhood. Twenty four percent of men had PTSD symptoms, 24% had depressive symptoms and 36% binge drank. Fifty six percent of men physically abused and 31% sexually abused partners at least once in their lifetime. Twenty two percent of men had one episode and 40% had repeat episodes of IPV perpetration. PTSD symptomatology risk increased with severity of child trauma and other trauma. PTSD severity increased the risk for binge drinking. Child trauma, other trauma and PTSD symptomatology increased the severity of depressive symptoms. PTSD symptomatology was comorbid with alcohol abuse and depressive symptoms. Child trauma, having worked in the year before the survey, other trauma and PTSD increased the risk of repeat episodes of IPV perpetration. Highly equitable gender attitudes were protective against single and repeat episodes of IPV perpetration. There was a direct path between the history of child trauma and IPV perpetration and three other indirect paths showing the mediating effects of PTSD, other trauma and gender attitudes.

**Conclusions:**

Child trauma is a risk factor for both poor mental health and male-perpetrated IPV among men in Gauteng. Male-perpetrated IPV in these settings should be explained through a combination of the Trauma, Feminist, and Intergenerational Transmission of Family Violence theories. Prevention interventions for male- perpetrated IPV in South Africa need to incorporate strategies and therapies to address poor mental health conditions.

## Introduction

Effects of child abuse and neglect in adults include persisting mental health problems such as personality disorders, PTSD, dissociative disorders, depression, anxiety disorders and psychosis [1,2]. PTSD, depression and alcohol abuse are comorbid and among the endemic mental health problems in South Africa [3–5]. The high prevalence of PTSD symptoms in the country is attributed to multiple violence exposures both in the pre and post-apartheid eras [4,6]. The violence exposures include child abuse, interpersonal and political violence throughout the life course [7–10].

The Trauma theory posits that life experiences of terror, violence and abuse whether in combat or at home are harmful and lead to psychological effects [11,12]. Victims of abuse therefore process their experiences as traumatic events similar to the response in individuals with post-traumatic stress [11]. Having PTSD is also a risk factor for other long-term and negative relational outcomes [13]. To date this theory has been substantiated by work among military veterans in developed countries and fewer studies have been conducted in general populations in low and middle income countries [14–16]. The body of knowledge showing a relationship between PTSD and male-perpetrated IPV in the general non-clinical populations is still emerging [17]

In South Africa, IPV (emotional or psychological, economic, physical and sexual violence) and rape are pervasive social problems. In a study conducted in the Eastern Cape and KwaZulu Natal Provinces, 28% of men reported rape perpetration and 42% of men reported being physically violent towards an intimate partner [18]. More recent studies reported lifetime prevalence of physical IPV perpetration of 19% to 33%; lifetime prevalence of sexual IPV perpetration of 5% to 19%; and lifetime prevalence of non-partner rape perpetration of 9% to 37% [19]. Research in South Africa has shown child abuse to be a risk factor for IPV perpetration [20–24]. To date, male-perpetrated IPV in South Africa has mostly been explained through Feminist theory which posits that IPV has its root in gender inequality and socialization that is the result of patriarchal societal norms in which men dominate over women.

The mediating role of mental health in the association between being a victim of child abuse and becoming an adult male IPV perpetrator has not been extensively investigated. It is critical to further elucidate the complex relationship in a South African setting where the three constructs (poor mental health, child abuse and IPV) overlap as public health concerns.

We have analysed data to further understand the relationship between child abuse, poor mental health and IPV perpetration in Gauteng province of South Africa in a sample of men from the general population. Our study obtained self-report data from adult males on traumatic events and other factors under conditions of complete confidentiality and anonymity. We aimed to test four hypotheses: first that child physical and sexual abuse experienced by men are traumatic events that increase the risk for PTSD, depression and binge drinking; secondly PTSD and other poor mental health conditions, due to child physical or sexual abuse or other traumatic experiences, increases the risk to aggression and partner abuse among male child abuse victims compared to men who were not victims; thirdly that PTSD and the poor mental health symptoms: alcohol abuse and depression mediate the relationship between experiencing child physical or sexual abuse and the risk of becoming an IPV perpetrator as an adult; and lastly that there is a direct relationship between child abuse history and IPV perpetration.

## Methods

In 2010, we conducted a cross-sectional population-based household survey of men aged 18 years and older in Gauteng province of South Africa. A two-stage sampling design was implemented. Seventy-five primary Sampling Units (PSUs) were randomly sampled and 20 dwelling points were selected per PSU. Within each dwelling point we randomly selected one household and within each household we selected one eligible participant. Researchers did not replace or substitute households or individuals if they did not have an eligible member or selected person was unavailable or unwilling to be interviewed. Ethics approval for this study was given by the University Of Witwatersrand Faculty Of Health Science Human Ethics Committee and the Medical Research Council Ethics Review Committee. The WHO Ethical and Safety Recommendations for Research on Domestic Violence against Women were followed in the study to ensure safety of the researchers and participants [25]. Participants gave their written informed consent to participate in the study. Researchers assured participants that their interview would be anonymous and data kept confidential. Interviews were conducted with 487 men who were mentally competent and stayed four nights a week or more in a selected household. Highly trained male interviewers administered the questionnaire in the participants preferred language i.e. English Zulu, Sotho or Afrikaans. Responses from the participants were captured using Personal Digital Assistants (PDAS). All interviews were conducted in private to achieve more open disclosure of sexual behaviours [26]. For the purpose of this study, data used were anonymous and did not contain any identifiable indicators.

### The questionnaire

The main outcome of the study was sexual or physical IPV perpetration. IPV was measured using the WHO Multi-country Study on Women’s Health and Domestic Violence: Core Questionnaire and WHO Instrument–Version 9 designed for use in developing countries [27]. Men were asked questions about perpetration of violence against female partners. Questions contained specific, objective descriptions of violent behaviour by men and asked about perceived frequency. Men were asked about perpetration of sexual violence against women who were not girlfriends, and also about their experiences of sexual victimization. Physical IPV perpetration included any of the following acts of violence (5 items): slapping, throwing dangerous objects, pushing, kicking, hitting, dragging, choking, beating, burning or threatening a current or previous intimate partner with a weapon. Sexual IPV perpetration included any of the following: physically forced non-consensual sex or sex because their partner was afraid of what he might do or forcing a partner to do something sexual that a partner found degrading or humiliating (3 items). For our analysis we created a combination variable that included physical and sexual IPV with the following categories: no sexual or physical IPV, sexual or physical IPV once and sexual or physical IPV more than once. We used this approach because previous research has shown overlap between sexual and physical IPV perpetration. Men who are physically violent towards their partners are at increased risk of risky sexual behaviours and to have raped [28]. We adopted this approach which has also been used in other research [29].

#### Child abuse

The main exposure variable was child abuse. We used a modified version of the short form of the Childhood Trauma Questionnaire (CTQ) (13 items, Cronbach’s alpha = 0.75) [30,31]. The CTQ had a four point Likert range of responses. Participants were asked whether they had experienced each act in their childhood. Possible responses were “never” (0), “sometimes” (1), “often” (2), or “very often” (3). For this study we created categorical variables for four dimensions of adversity namely physical abuse and punishment; sexual abuse; emotional abuse; or emotional neglect (**Table 1**). We also created a continuous variable of child trauma by summing up the scores from all the CTQ item responses. We used the continuous child trauma score in regression and SEM model analyses.

**Table 1:** Childhood trauma dimensions and scale items.

***Before I reached 18*:**	
*Emotional neglect-*	*Emotional abuse*
I did not have enough to eat.	I saw or heard my mother being beaten by her husband or boyfriend.
I lived in different households at different times.	I was insulted or humiliated by someone in my family in front of other people.
One or both of my parents were too drunk to take care of me.	I was told I was lazy or stupid or weak by someone in my family.
I spent time outside the home and none of the adults at home knew where I was.	
*Sexual abuse*	*Physical abuse*
Someone touched my buttocks or genitals or made me touch them when I did not want to.	I was beaten at home with a belt or stick or whip or something else which was hard.
I had sex with a woman who was more than 5 years older than me.	I was beaten so hard at home that it left a mark or bruise.
I had sex with someone because I was threatened or frightened or forced.	I was beaten or physically punished at school by a teacher.
***Gender Equitable Men Scale***	
You don’t talk about sex, you just do it.	Men are always ready to have sex.
There are times when a woman deserves to be beaten.	I would be outraged if I asked him to use a condom.
Changing nappies, giving kids a bath, and feeding the kids are the mother’s responsibility.	If someone insults me, I will defend my reputation, with force if I have to.
It is a woman’s responsibility to avoid getting pregnant.	To be a man, you need to be tough.
A man should have the final word about decisions in his home.	Men should be embarrassed if they are unable to get an erection during sex.

#### Poor mental health

In this study we hypothesized that poor mental health has a mediating effect in the relationship between child abuse (exposure) and adult IPV perpetration (outcome). We measure poor mental health outcomes namely PTSD, depression and alcohol abuse. PTSD was measured using the Harvard Trauma Questionnaire (HTQ) which has been validated in other populations and cultural settings (30 items, Cronbach’s alpha = 0.95). Respondents were asked whether they were bothered by each symptom in the past weeks and rated on a four point Likert scale: “not at all” (0), “a little” (1), “quite often” (2) and “extremely often” (3). Scores for each respondent that responded to all the items were summed to give a PTSD scale score. Only seven percent of men scored above the critical cutoff of 75 that is used for PTSD screening in refugee populations [32–34]. Because we are unaware of any previous studies validating cut off points of the HTQ in a general South African population sample we considered a conservative score equal or greater than 25 as being ‘checklist-positive’ for PTSD.

Depressive symptoms were measured using the 20-item Center for Epidemiologic Studies Depression (CESD) self-report measure which was designed for use in the general population to assess current symptoms of depression (Cronbach’s alpha = 0.86) [35]. The CESD scale assesses how often an individual experienced symptoms associated with depression over the past week. The items were responded to on a four point Likert scale, with response categories ranging from “rarely or none of the time” (0) to “most or all of the times” (3) which we summed up to a total CESD score. Higher scores indicated more severe depressive symptoms (range of possible scores 0 to 60). We used the CESD scale as a continuous scale and as a dichotomous variable with a cut-off score of 16. Previous research in South Africa have used the 16+ as a cut-off point for probable depressive symptoms in community-based research studies [36,37]

We used two items of the Alcohol Use Disorders Identification Test (AUDIT) scale [38] to assess for hazardous alcohol use. We assessed item 1: How often do you have a drink containing alcohol? Responses were never(0), monthly or less (1), 2–4 times a month (2), 2–3 times a week (3), 4+ times a week (4).We also assessed item 3: How often have you had 5 or more drinks on one occasion in the last year. Responses were: never (1), less than monthly (2), monthly (3), weekly (4), and daily or almost daily (5). The measures are part of AUDIT C (first three questions on the Audit scale). For regression analysis we confined measurement of alcohol to AUDIT-3 (third question). We considered a score of 4 or more (equivalent to drinking 5 or more drinks on one occasion weekly or daily) as binge or hazardous drinking. Several studies have found that both the AUDIT-C and AUDIT-3 are comparable to the original AUDIT across various settings and different racial or ethnic groups [39–42]

#### Other trauma

We also measured other trauma exposures through an adapted Life Event Checklist from the PTSD Checklist (10 items). Participants were asked whether they had experienced any of the following traumatic events imprisonment/detainment, civil unrest/war, serious injury requiring hospitalization, being close to death, witnessing a murder of family or friend, unnatural death of family or friend, witnessing the murder of stranger/s, torture, robbed or carjacked at gun or knife point and kidnapping. Participants had to respond for each of the checklist items with yes (1) and no (0).responses. For this analyses we summed the scores for the items including witnessing murder of someone close, stranger murder, torture, death, and having been kidnapped to create a continuous “other trauma exposure” variable. A greater score was indicative of more lifetime traumatic events or exposures. Because other traumatic exposure is a known risk factor for PTSD we adjusted for this continuous variable in all regression analysis with a PTSD outcome. We created another dichotomous trauma events exposure with a score of zero being no other trauma exposure and a score of one or more being “other trauma exposure”. We used the dichotomous variable in descriptive cross tabulation methods.

Risk factors identified in previous research and collected in the survey included the socio-demographics age, education, employment status and number of sexual partners in the year before the study. To measure masculinities and gender attitudes, we used the Gender Equitable Men Scale (GEMS) (10 items, Cronbach’s alpha = 0.71) (**Table 1**). We categorized the GEMS score into three categories: low, neutral and high based on tertiles of the distribution.

### Statistical analysis

All data analyses was conducted in Stata 13 accounting for the stratified, two-stage survey design with participants clustered within the PSUs. We cleaned the data set and excluded men that had never been in an intimate relationship or who had missing data on key variables (n = 71: 23 cases of men who were never in an intimate relationship, 15 cases with missing data on child trauma variables, 1 case with missing data on the CESD variables, 1 case with missing data on the PTSD variables, 31 cases with missing data on IPV variables). We recoded data to create the child abuse, poor mental health and IPV categorical variables.

We assessed internal consistency of all the scales by evaluating the Cronbach’s alpha. We conducted bivariate analyses of the outcome variable against the exposure variable and other selected socio-demographic variables. We conducted bivariate analyses and reported on results as percentages together with the Pearson chi squared statistic test for differences in proportions. We used the 0.05 level of significance as a reference (**Table 2**)**.**

**Table 2:** Characteristics of men who perpetrated IPV.

	SEXUAL IPV NEVER		SEXUAL IPV ONCE OR MORE			PHYSICAL IPV NEVER		PHYSICAL IPV ONCE OR MORE			NO SEXUAL /PHYSICAL IPV		ONE EPISODE OF S/P IPV		MORE THAN ONE EPISODE OF S/P IPV		
	N	%	N	%	P value	N	%	N	%	P value	N	%	N	%	N	%	P value
***Age group***																	
18–29 years	105	36.6	53	41.1	0.08	74	40.7	84	35.9	0.1	60	38	38	24.1	60	38	0.3
30–44 years	95	33.1	52	40.3		54	29.7	93	39.7		47	32	32	21.8	68	46.3	
45+ years	87	30.3	24	18.6		54	29.7	57	24.4		50	45.1	23	20.7	38	34.2	
***Education***																	
High school incomplete and lower	137	47.7	56	43.4	0.5	72	39.6	121	51.7	0.01	68	35.2	41	21.2	84	43.5	0.3
High school complete and higher	150	53.3	73	56.6		110	60.4	113	48.3		89	40	52	23.3	82	36.8	
Employed in past 12 months	193	67.3	90	69.8	0.7	114	62.6	169	72.2	0.02	100	35.3	62	21.9	121	42.8	0.3
***In an intimate relationship***	255	88.9	124	96.1	0.01	163	89.6	216	92.3	0.4	141	37.2	84	22.2	154	40.6	0.6
***Child abuse***																	
Any child physical abuse	252	87.8	114	88.4	0.9	150	41	216	59	0.009	132	36.1	81	22.1	153	41.8	0.1
Any child sexual abuse	52	18.1	33	25.6	0.1	29	34.1	56	65.9	0.05	21	24.7	24	28.2	40	47.1	0.04
Any child emotional abuse	171	59.6	94	72.9	0.03	96	52.8	169	72.2	<0.001	85	54.1	51	54.8	129	77.7	<0.001
Any child emotional neglect	162	56.5	69	53.5	0.5	99	54.4	132	56.4	0.8	87	55.4	46	49.5	98	59	0.4
***Other trauma exposures (> = 1)***	248	86.7	115	90.6	0.2	146	80.2	217	93.9	<0.001	126	80.3	81	88	156	95.1	0.004
***Mental health***																	
Binge drinking	108	37.6	40	31	0.2	66	44.6	82	55.4	0.9	54	36.5	40	27	54	36.5	0.3
Depression (> = 16)	56	19.5	42	32.6	0.01	32	17.6	66	28.2	0.004	26	14.7	49	27.6	49	29.5	0.01
PTSD (> = 25)	54	18.8	44	34.1	0.003	29	15.9	69	29.5	0.001	23	23.5	23	23.5	52	53.1	0.005
**Gender equitable norms**																	
Low	24	8.3	15	11.6	0.5	8	4.4	31	13.3	0.004	6	15.4	14	35.9	19	48.7	0.005
Neutral	172	59.9	77	59.7		105	57.7	144	61.5		87	34.9	56	22.5	106	42.6	
High	91	31.7	37	28.7		69	37.9	59	25.2		64	50	23	18	41	32	
**Total**	**287**	**69**	**129**	**31**		**182**	**43.8**	**234**	**56.3**		**157**	**37.7**	**93**	**22.4**	**166**	**40**	

To measure the association between child abuse exposure and IPV outcome we used the three category compound variable for IPV perpetration: no sexual or physical IPV; one episode of sexual or physical IPV only; and more than one episode or type. The child physical and sexual abuse variables were dichotomous (never vs ever). We conducted bivariate logistic regression models to test the association between child abuse variables and the poor mental health outcomes (**Table 3**). We then conducted multinomial regression analysis to test child abuse variables as risk factors for sexual or physical IPV perpetration while adjusting for poor mental health variables and other potential confounding variables that had a significance < = 0.3 in bivariate analysis (**Table 4**). We used a stepwise backwards elimination to drop variables with non-significant p-values until the models appeared parsimonious. We report on the odds ratios and the alpha statistic at the significance level of 0.05.

**Table 3:** Associations between poor mental health outcomes, child abuse and other violence exposures.

	PTSD		Binge drinking		Depressive symptoms	
	OR (95% CI)	P value	OR (95% CI)	P value	OR (95% CI)	P value
Child trauma score	1.1 (1.1; 1.2)	<0.001	1.1 (0.9: 1.0)	0.5	1.2 (1.1;1.3)	<0.001
Other trauma exposures	8.5(1.8;39.2)	0.007	1.5 (0.8;3.0)	0.2	1.5(1.2;1.7)	<0.001
PTSD			1.7(1.0;2.7)	0.04	12.5(6.9;22.8)	<0.001
Binge drinking					1.3(0.7;2.4)	0.7

**Table 4:** Multinomial regression model for IPV perpetration.

	One episode of Physical /sexual IPV perpetration		More than one episode of Physical /sexual IPV perpetration	
	AOR (95% CI)	P value	AOR (95% CI)	P value
Child trauma score	1.0 (0.9;1.1)	0.9	1.1 (1.0;1.2)	0.02
Worked in 12 months before survey	1.3 (0.7;2.4)	0.4	1.9 (1.1;3.4)	0.02
PTSD score	1.0 (1.0;1.1)	0.1	1.03 (1.0;1.1)	0.02
Depression score	1.0 (1.0;1.1)	0.7	1.0 (1.0;1.1)	0.9
Binge	1.2 (0.7;2.1)	0.5	0.8 (0.5; 1.4)	0.4
Other trauma exposures	1.2 (0.9;1.7)	0.1	1.6 (1.2;2.1)	<0.001
Low inequitable gender attitudes	1.0		1.0	
Mid-level gender attitudes	0.3 (0.1; 0.9)	0.03	0.6 (0.2;1.7)	0.3
Highly equitable gender attitudes	0.2 (0.1;0.5)	0.002	0.3 (0.1;0.8)	0.02

We conducted Structural Equation Modelling (SEM) with maximum likelihood estimation to test pathways/structural relationships between child abuse, poor mental health and sexual or physical IPV perpetration. First we determined the appropriate latent factors for child abuse and poor mental health and IPV perpetration through fitting separate measurement models. We then fit models to test the relationship of child trauma (as a latent factor), the poor mental health dimensions (as separate observed variables) and sexual or physical IPV perpetration (also as observed variable). We included the gender attitudes variable (observed) to adjust for the relationship between child trauma and IPV perpetration; and other traumatic exposures (observed) to adjust from the relationship between child trauma and PTSD symptomatology. We also adjusted the model by adding the employment status variable (observed). We tested the fit of the model by assessing the Comparative Fit Index and the Root mean squared error of approximation (RMSEA). The standardized parameter estimates are displayed in the model and tabulated (**Table 5**). The data fit the model well and the fit indices are shown in **Table 6.**

**Table 5:** Structural model statistics.

	Coefficient (β)	P value
**Gender attitudes**		
➢ Child trauma score	-4.9	0.01
**Other trauma score**		
➢ Child trauma score	2.0	<0.001
**PTSD**		
➢ Other traumatic exposure	3.5	<0.001
➢ Child trauma score	18.3	0.003
**Depression**		
➢ PTSD	0.4	<0.001
➢ Child trauma	4.6	0.01
**Binge drinking**		
➢ Depression	-0.1	<0.001
➢ PTSD score	0.07	<0.001
Worked		
➢ PTSD score	-0.003	0.04
**IPV perpetration**		
➢ Gender attitudes	-0.04	<0.001
➢ Child trauma score	0.7	0.02
➢ PTSD	0.01	<0.001
➢ worked	0.2	0.02
➢ Other traumatic exposure	0.1	<0.001

**Table 6:** Model Goodness of Fit statistics.

Fit statistic	Value
*Likelihood ratio*	
p > chi2	<0.001
*Population error*	
RMSEA	0.045
*Baseline comparison*	
CFI	0.943
TLI	0.912

## Results

The study sample included 416 men above the age of 18 and had 77% response rate. The majority of men (38%) were aged 18–29 years, 35% were aged 30–44 and 27% were above 45 years. Over half the men had completed high school education (54%), 68% were employed in the year before the survey. Ninety-one percent of men were in current relationships at the time of the survey and 45% had two or more sexual partners in the year preceding the survey. As children, 80% of men were physically abused, 55% were neglected, 63% were emotionally abused and 20% were sexually abused. Twenty five percent of men had witnessed the abuse of their mother. Three quarters of men (75%) drank alcohol in the year preceding the survey, 36% binge drank weekly or daily, 24% had depression symptoms and 24% had PTSD symptoms. Thirty one percent of men perpetrated sexual IPV and 56% perpetrated physical IPV in their lifetime. Twenty two percent of men had perpetrated sexual or physical IPV on one occasion and 40% perpetrated sexual or physical IPV on more than one occasion.

**Table 2**shows the characteristics of men by IPV perpetration group. Fewer men who had perpetrated physical IPV had completed high school, but schooling otherwise did not vary across groups. Men who perpetrated physical IPV were more often employed than those who didn’t but employment did not vary with sexual IPV perpetration. Men in current relationships more often reported sexual IPV perpetration compared to those not in relationships at the time of the survey. More men that were physically or sexually abused as children, perpetrated physical IPV compared to men who had not been victimised but there was no difference in sexual IPV perpetration. More men who had PTSD or depressive symptoms perpetrated sexual or physical IPV at least once in their lifetime.

**Table 3**shows associations between child trauma (continuous variable inclusive of all child abuse dimensions) and the poor mental health conditions. PTSD was associated with child trauma, other traumatic exposures, binge drinking and depressive symptoms. Depressive symptoms were also associated with child trauma and other traumatic exposures

**Table 4**shows that having mid-level gender attitudes [AOR 0.3; 95% CI (0.1; 0.9)] or highly gender equitable attitudes [AOR 0.2; 95%CI (0.1; 0.5)] (compared with very inequitable attitudes) was protective to one episode of IPV perpetration. Experience of child trauma and other traumatic events, having PTSD, and having been employed were associated with repeat episodes of IPV perpetration. High equitable gender attitudes were protective against repeated IPV perpetration. The more severe the childhood trauma, other trauma and PTSD symptoms, the higher the risk of repeated IPV perpetration.

**Fig 1:**
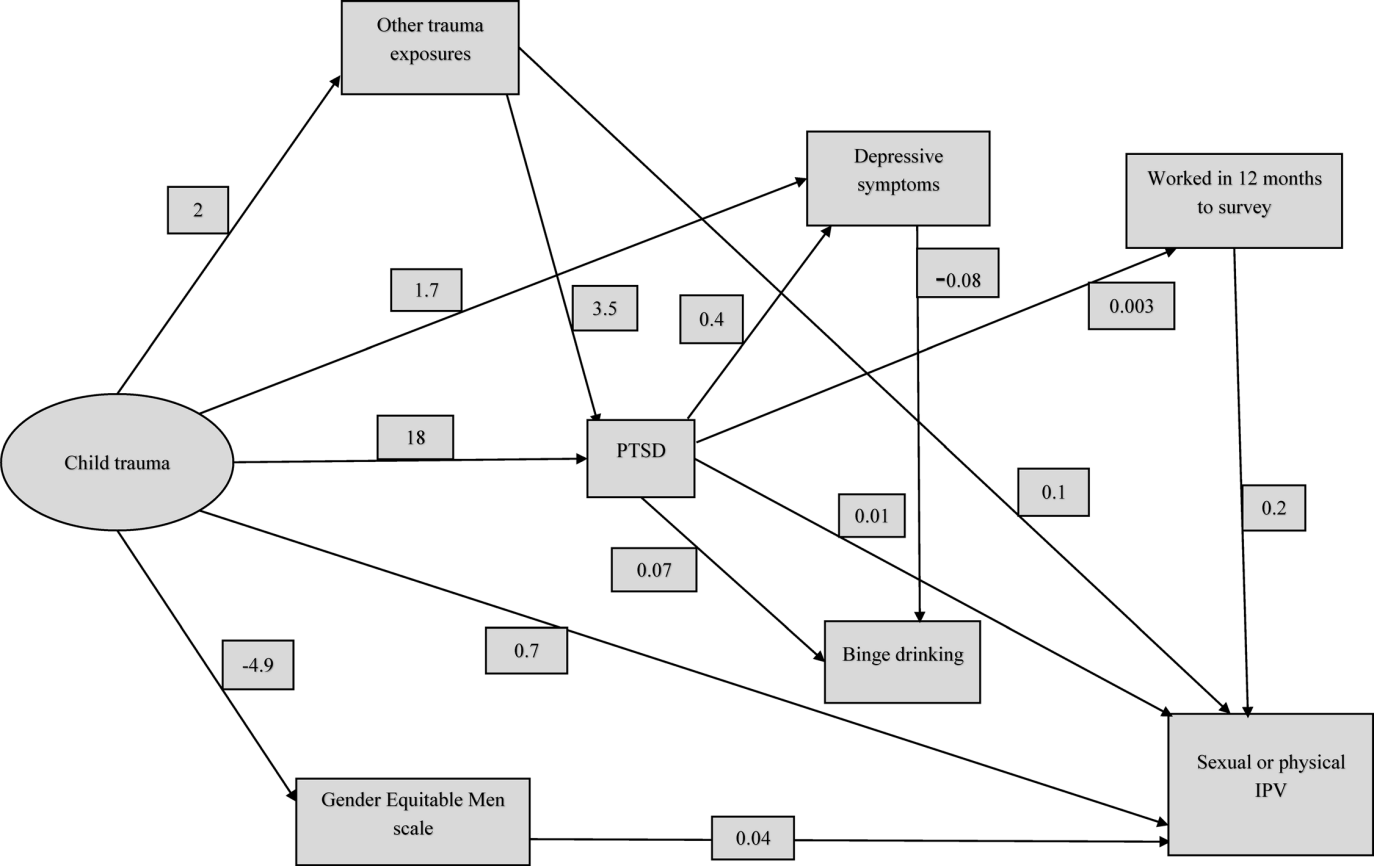
Structural model of IPV perpetration and poor mental health.

**Fig 1, Tables 5 and 6**show the final SEM model estimating the associations between child abuse, poor mental health and sexual or physical IPV perpetration by men while adjusting for gender attitudes, other trauma exposures and employment. There is a direct path from childhood trauma to IPV perpetration. The relationship is also mediated by PTSD, other trauma and gender attitudes. PTSD mediates the relationship of child trauma with depressive symptoms; and other trauma with IPV perpetration. The relationship of PTSD and binge drinking is mediated by depressive symptoms. There are no paths from depressive symptoms or binge drinking to IPV perpetration. There is also a path from PTSD to IPV mediated by employment status.

## Discussion

Our study contributes empirical evidence to the limited body of knowledge on the pathways between child abuse, poor mental health and IPV perpetration among a representative sample of men in South Africa. The study confirms overlap between poor mental health, history of child abuse and adult IPV perpetration which constitute public health problems. In this study an increase in child trauma was associated with an increase in risk of other trauma, PTSD, depressive symptoms and repeat IPV perpetration. PTSD and depressive symptoms were associated with child trauma in previous studies with South African youths.

An increase in PTSD score was associated with greater risk for binge drinking and depressive symptoms. The relationship of PTSD and depressive symptoms was however stronger compared to the relationship with binge drinking. While the study showed comorbidity of PTSD with depressive symptoms and binge drinking within the population, it found higher prevalence of these conditions compared to previous national surveys and this is attributable to measurement differences [5,7,10].

The study however did not find an association between child or other trauma and binge drinking. There was also no significant association between depression or binge drinking and IPV perpetration. In this regard our findings differ from previous studies showing binge drinking or alcohol abuse as a risk factor for IPV perpetration [21,43–45]. Nonetheless the findings are consistent with findings from a global meta-analytic by Foran et al 2007 that indicated small to moderate effect size for the association between alcohol abuse and male-perpetrated IPV with lesser or no association being found in non-clinical samples [46]. Previous research has also shown no differences in size of effect when the full Audit scale or shorter version have been used therefore it is difficult to conclude whether our observation is a result of the restrictive use of the Audit-3 binge drinking measure [39,40]

The structural model for the data mostly conforms to our priori-hypotheses. Child trauma increased PTSD and depressive symptomatology; PTSD increased risk of IPV perpetration; PTSD mediated the relationship between child trauma and IPV perpetration and there was a direct relationship of child trauma and IPV perpetration. These findings confirm that IPV perpetration in the studied population is explained by a hybrid of the known violence theories and emerging in this study is the contribution of the Trauma theory within the South African context. The direct path from child trauma to IPV perpetration which had the greatest co-efficient in this study is consistent with previous studies that reported that men who experienced physical abuse during childhood or were exposed to parental violence are at greatest risk of perpetration [20,47,48]. The direct path from child trauma to IPV perpetration is also consistent with the Intergenerational Transmission of Family Violence theory while the mediated path with gender attitudes conforms to the Feminist theory that has its roots in gender inequality and conservative gender norms [49,50]. The mediation effect of PTSD in the relationship of child or other trauma and IPV perpetration aligns to the Trauma theory which specifies an interconnection of violent experiences throughout life. According to the Trauma theory, abused boys more often are exposed to other forms of violent trauma and PTSD symptomatology and are at greater risk of aggression and partner abuse. In this study we found a mediating effect of PTSD in the paths between child or other trauma exposures and IPV perpetration consistent with other studies in different settings [24,51,52]. Moreover other trauma exposures were a stronger predictor for PTSD and repeat IPV perpetration than child trauma consistent with previous South African studies [7,8,53–56].

The mediating effects of PTSD in the relationship of child trauma and IPV perpetration by men suggests expansion in the understanding of violence in the South African context beyond the Intergenerational Transmission of Family Violence and the Feminist theories of patriarchal influences. These findings warrant further investigation to understand the differences in patterns and dynamics of IPV associated with PTSD and non-PTSD related IPV. The differences may highlight opportunities for potential interventions and have implications for the design of perpetrator programmes.

Our findings point to the need for IPV perpetration interventions to incorporate validated, appropriate and targeted psychotherapeutic approaches. They also give emergent evidence that can be basis for further research and baseline programme evaluations that assess whether implementation of current treatment therapies for PTSD among male IPV perpetrators could reduce both PTSD symptoms and IPV perpetration in this context [17]. Therapies for PTSD and depressive symptoms have been shown to reduce IPV perpetration in other settings [57,58].

The central position of PTSD in the pathways to IPV perpetration spell out the potential contributions of health professionals to a public health approach to responding to child trauma and in secondary prevention of violence. The evidence from this study supports the need to develop integrated primary and secondary prevention interventions that incorporate mental health components which build emotional resilience and coping mechanisms of participants. This may reduce the psychological impact of past trauma while preventing future violence perpetration among previously victimised boys and men. The collaboration of health practitioners and researchers in the field is critical in the design of future integrated psychosocial treatments that take a holistic approach and emphasize addressing mental health at the individual, family and societal levels [59]. Another potential entry for prevention are psychosocial interventions in school based programmes with educator and learner participation that include stress modules and coping strategies which have been effective in other settings [59].

Notwithstanding, these findings underscore the need for development of more positive parenting interventions which break the intergenerational transmission of violence in this context. Parenting interventions that discourage physical beating as a punitive measure while not necessarily reducing PTSD symptomatology may potentially reduce the incidence of physical IPV perpetration in the next generation. In South Africa concerted efforts to discourage child maltreatment could contribute positively in the fight against partner abuse.

The study had limitations. Its relatively small sample size limited statistical power to detect small associations and secondly has the potential for residual confounding consistent when investigating a complex outcome such as IPV. This study was unable to gauge the chronicity of the PTSD reported by participants. Chronicity of PTSD could have impacted on the risk for IPV perpetration [60]. Additionally there is ambiguity in direction of variables because the study is cross-sectional. The cross-sectional design has the limitation of not elucidating the temporal relationship between PTSD and IPV perpetration. Further research which employs longitudinal studies with a male cohort on the relationship is critical, and might be strengthened by biological indicators of mental health outcomes where available such as cortisol and alcohol abuse. Longitudinal prospective studies with cohort of abused boys and controls may also shed further light into the effect of chronicity of PTSD on IPV perpetration. The validation of cut-off points for CESD, PTSD and Audit-3 as a measure in the South African context is also critical in strengthening the quality of research. The study also did not adjust for several mental health related responses including insecure attachment, borderline personalities, hyper arousal and anger responses, which were found to mediate pathways to IPV in previous research. However the study provides a basis for the inclusion of mental health variables in future large scale IPV perpetration surveys, to further investigate the relationship suggested by this study. Beyond understanding male-perpetrated IPV, increasing the mental health data is critical and has good value for money in a region like Africa where research on mental health is still limited.

## Conclusion

The study confirmed a relationship between child trauma and IPV perpetration, which was partially mediated by PTSD. Evidence from this empirical study suggests a nexus of the Trauma, Feminist and Intergenerational Transmission of Family Violence theories in explaining the underlying factors why men perpetrate violence against intimate partners. The study showed that PTSD associated with childhood sexual abuse and other traumatic events increased risk for male-perpetrated sexual or physical IPV. A direct path from child physical adversity and physical IPV perpetration in this context was consistent with the Intergenerational Transmission of Family Violence theory. Reducing risk of trauma exposure due to abuse or witnessing family violence may impact on reduction of PTSD and male-perpetrated IPV. Pharmacotherapeutic and psychotherapeutic interventions for treating PTSD symptoms among men or boy sexual abuse victims may have the potential to reduce male-perpetrated IPV. There is need for future large scale research to understand the contribution of a wider range of poor mental health conditions to male-perpetrated IPV perpetration using validated tools in the African context.
